# Muscle energy technique for chronic obstructive pulmonary disease: a systematic review

**DOI:** 10.1186/s12998-019-0256-9

**Published:** 2019-08-20

**Authors:** Danielle A. Baxter, Johannah L. Shergis, Azharuddin Fazalbhoy, Meaghan E. Coyle

**Affiliations:** School of Health and Biomedical Sciences, RMIT University, PO Box 71, Bundoora, VIC 3083 Australia

**Keywords:** Manual therapy, Muscle energy technique, COPD, Systematic review

## Abstract

**Background:**

Chronic Obstructive Pulmonary Disease (COPD) is an increasingly prevalent respiratory disease that impacts on daily living. In addition to difficulty breathing, many people experience extrapulmonary comorbidities such as musculoskeletal disorders. Pulmonary rehabilitation can improve fitness and strength but may be difficult for patients with musculoskeletal disorders. Recent research indicates promising benefits of adding manual therapy to standard care to improve clinical outcomes.

**Objectives:**

To evaluate the efficacy and safety of Muscle Energy Technique (MET) for people with COPD.

**Methods:**

Ten databases were searched from inceptions to May 2018. Eligible studies were randomised controlled trials assessing MET compared to any control for COPD. Outcomes included lung function, exercise capacity, health-related quality of life, and adverse events.

**Results:**

Three randomised controlled trials assessing 90 participants were included. The quality of the research was limited by reporting of outcome measures and results, varying treatment protocols, and small sample sizes. Results from one study showed that pulmonary function was not statistically different between groups at end of treatment (FEV_1_% MD 4.87%; 95% CI − 0.79 to 10.53). Exercise capacity and perceived dyspnoea ratings were improved in single studies. Adverse events were unrelated to the MET intervention.

**Conclusions:**

The use of MET for COPD is an emerging field of research, with few studies evaluating its efficacy and safety. Currently, there is insufficient evidence to support the use of MET in the management of COPD. Rigorously designed studies with larger sample sizes are needed to better understand the role of MET for COPD.

**Electronic supplementary material:**

The online version of this article (10.1186/s12998-019-0256-9) contains supplementary material, which is available to authorized users.

## Background

Chronic Obstructive Pulmonary Disease (COPD) is a progressive condition which is characterised by ‘chronic obstruction of lung flow that interferes with normal breathing and is not fully reversible’ [1]. Currently there is no cure for COPD. International guidelines [2, 3] recommend that treatment and management of COPD should be individualised to manage symptoms, reduce the risk of exacerbations, improve quality of life and exercise tolerance. An integrated multidisciplinary approach to management is required, including pharmacotherapy and pulmonary rehabilitation [4].

Although COPD is primarily a disease of the lungs, the involvement of associated extrapulmonary comorbidities have been recognised in recent years [2, 5]. This includes musculoskeletal disorders such as skeletal muscle dysfunction, osteoporosis, muscle loss, [6] along with an increased prevalence of cervico-thoracic pain [7]. Additionally, mechanical restriction is thought to be one of the causes of activity-limiting dyspnoea [8, 9] and postural adaptations may also be associated with reduced pulmonary function [10]. Manual therapy may have a role to play in the management of COPD by utilising manual techniques to address the musculoskeletal dysfunction [11–13]. Clinical studies have evaluated the use of spinal manipulation, [14, 15] myofascial release techniques, [16] soft tissue techniques [17] and osteopathic manipulative treatments [18, 19] to address musculoskeletal disorders in COPD patients with varying results.

Muscle Energy Technique (MET) is one such manual therapy that has been used to treat COPD. MET is a gentle technique used in clinical practice by a wide range of manual therapy practitioners, including physiotherapists and osteopaths [20–22]. It is commonly used to treat hypertonic muscles and to improve joint mobility [23, 24]. The isometric version of the technique is most commonly employed and involves specific components: 1. Localisation of joint/muscle barrier by operator (controlled joint positioning); 2. Patient active muscle contraction in a specific direction for a specified time; 3. Operator-applied distinct counterforce against the patient contraction; 4. Patient relaxation; 5. Operator re-uptakes the ‘new’ barrier (passive stretch of the muscle, or increase in joint movement in a specific direction); 6. Repeat procedure several times [24]. Research has shown that MET may increase muscle flexibility, [25, 26] spinal range of motion [27–30] and shoulder joint range of motion [31, 32]. The physiological mechanisms that underpin MET are unclear. However, it is thought to act through a complex interplay of neurophysiological mechanisms which have an effect on tissue extensibility and tolerance due to pain modulation [23, 33]. It is thought that the application of MET to the thoracic cage and associated musculature may aid in improving the mechanical restrictions commonly seen in people with COPD, which could further impact outcomes such as dyspnoea, exercise capacity and pulmonary function.

This systematic review evaluates the efficacy of MET for people with COPD in terms of lung function, exercise capacity, dyspnoea and quality of life. In addition, the safety of MET is assessed.

## Methods

We followed the methods described in the Cochrane Handbook of Systematic Reviews [34] and registered the review in PROSPERO (ID No. CRD42017070076). We have reported items according to the PRISMA checklist (see Additional file 1).

Randomised controlled trials (RCTs) published in English were included. Participants were adults aged 40 years or over with a diagnosis of COPD according to the Global Initiative for Chronic Obstructive Lung Disease (GOLD) criteria [2]. Studies of participants with respiratory illnesses other than COPD were excluded. Included interventions were MET or a similarly described technique. This includes a manual therapy technique applied by an external operator to a joint or muscle, which involves both an active patient muscle contraction and passive movement from the operator which is repeated for a specified number of times. Studies were considered eligible if the technique described was applied in a similar manner to MET, even if a different name was used. For example, Proprioceptive Neuromuscular Facilitation (PNF) stretching involves active muscle contraction by the patient followed by a passive stretch by the operator. This aspect is common to both MET and PNF. All types of control interventions were eligible, including no treatment, sham treatment or treatments recommended in clinical practice guidelines.

The primary outcomes were measures of pulmonary function and capacity, including inspiratory capacity, forced expiratory volume in one second (FEV_1_), and forced vital capacity. Secondary outcomes included exercise capacity measured by the six-minute walk test (6MWT), quality of life or health status measured by validated questionnaires, for example the COPD Assessment Test, St. George’s Respiratory Questionnaire, and the Chronic Respiratory Questionnaire. Safety was assessed by reviewing adverse events.

A literature search was conducted in ten electronic databases from their inceptions to August 2017. Databases included the Cochrane Central Register of Controlled Trials (CENTRAL), Pubmed, Embase, Cumulative Index to Nursing and Allied Health Literature (CINAHL), Allied and Complementary Medical Database (AMED), Scopus, Physiotherapy Evidence Database (PEDro), Index to Chiropractic Literature, Osteopathic Medicine Digital Repository (OSTMED-DR), and Osteopathic Research Web. Key search terms were related to COPD (*Chronic Obstructive Pulmonary Disease*, *COPD*, *bronchitis* and variants); MET (*muscle energy technique*, *MET*, *post-isometric contraction* and variants), and randomised controlled trials (*randomized controlled trial*, *controlled clinical trial* and variants). An update search was conducted on 24th May 2018 to identify any additional studies published since the previous search.

Two review authors (DB, MC) independently screened titles and abstracts of the results identified in the search. The full texts of potentially eligible studies were read and independently evaluated for inclusion. Disagreement between evaluations was resolved through discussion. Two review authors (DB, MC) independently extracted study data to ensure accuracy. The following data were extracted to a pre-defined data extraction form: characteristics of the study including participants, intervention, comparator, and results. Attempts were made to collect missing data from study authors via email. If no response was received after two weeks, the data was marked as ‘not available’ and excluded from analysis. This action was taken for all missing data as no response was received from study authors.

Data was analysed using risk ratios (RR) and 95% confidence intervals (95% CI) for dichotomous data and mean difference (MD) with 95% CI for continuous data. Meta-analysis using a random effects model was planned. Exploration of substantial statistical heterogeneity through sub-group analysis was also planned where the Chi square test was less than 0.10 and I^2^ statistic was greater than 50%; planned sub-groups included treatment duration, treatment frequency, and stage of disease. A sensitivity analysis was also planned to include studies judged as low risk of bias for sequence generation. However, due to the small number of included studies, planned sub-group analyses were not possible. Risk of bias was assessed using the Cochrane Collaboration Risk of Bias Tool [34]. Assessment was made independently by two researchers (DB, MC), and disagreements were resolved through consultation with a third reviewer (JS). All domains were scored as either low, high, or unclear risk of bias.

## Results

**Fig. 1:**
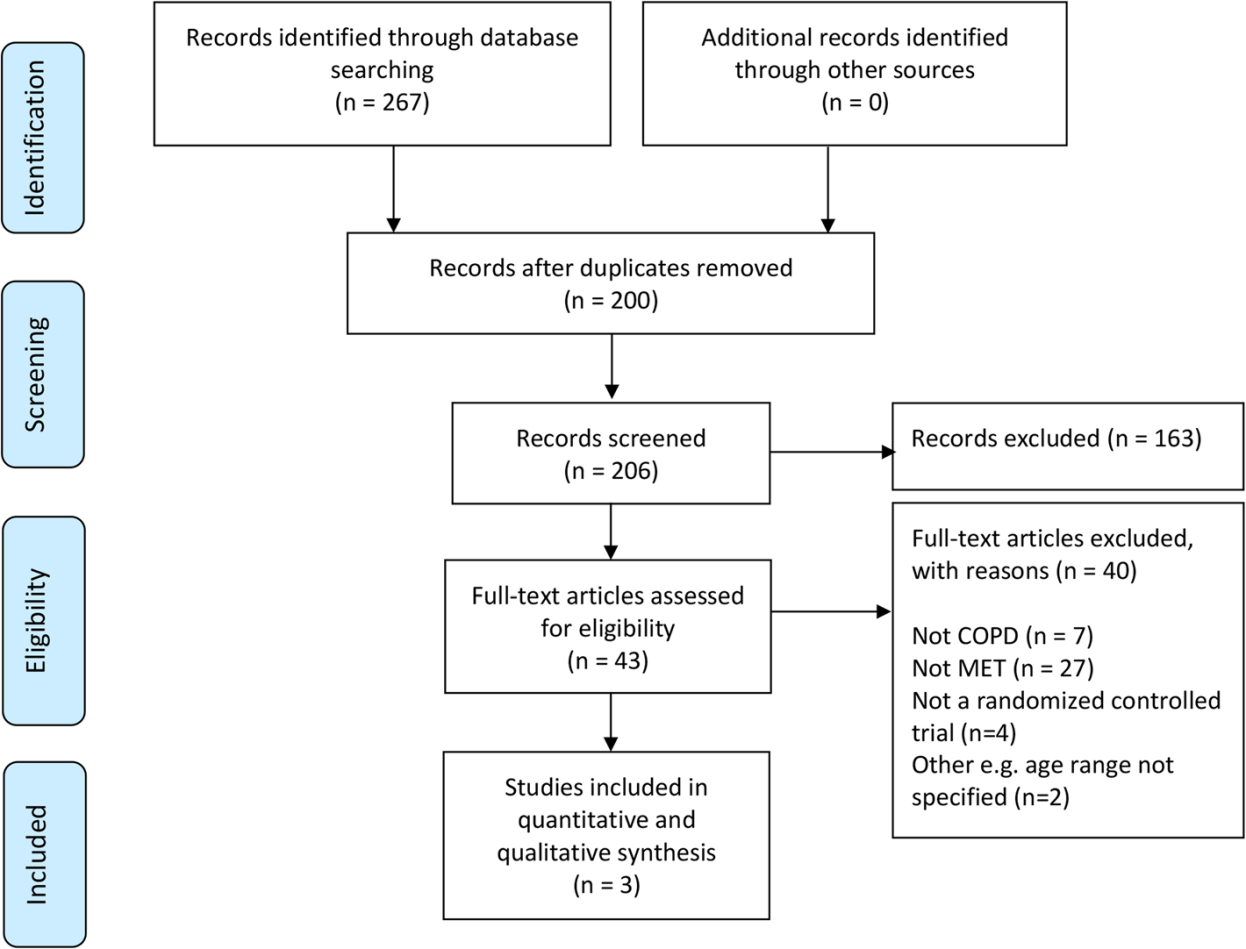
Study selection flow chart

The database search identified 267 potentially relevant citations. After duplicates were removed, 206 citations were screened and 163 were excluded. The full text articles of 43 studies were reviewed. Forty articles were excluded leaving three studies (90 participants) included (Fig. 1). Characteristics of included studies are outlined in Table 1. One study did not specify the number of participants allocated to the intervention and control groups [35]. For this study it was assumed randomisation resulted in equal group numbers for the purpose of analysis. One study was conducted in India, [35] one in Brazil [36] and the location of the third study was unspecified [37]. All trials were published in English.

**Table 1:** Characteristics of the included studies

First author, publication year	No. randomised, (dropouts, if applicable)	Age in years (mean and SD or range)	Treatment duration; No. of treatments	Intervention protocol	Comparator	Outcome measures
Anand 2013 [37]	I: 15 (NS) C: 15 (NS)	40–70	3 days; 3	MET + CPT	CPT	6MWT CCQ Borg scale
Sule 2017 [35]	Total: 30 (NS)	40–60	NS; NS	MET	Sham + CPT	FEV_1_
Wada 2016 [36]	I: 15 (1) C: 15 (1)	I: 61 (5.4) C: 64 (5.6)	12 weeks; 24	MET + exercise	Sham + exercise	6MWT Modified Borg scale

Abbreviations: *CCQ* Clinical COPD Questionnaire, *C* control group, *CPT* conventional chest physiotherapy, *FEV*_*1*_ forced expiratory volume in one second, *I* intervention group, *MET* muscle energy technique, *NS* not specified, *SD* standard deviation, *6MWT* six-minute walk test

The studies were published between 2013 and 2017. In all three studies, participants were classified as having moderate to severe COPD. Two studies [35, 37] compared MET with standard conventional chest physiotherapy (CPT) exercises, including breathing and thoracic expansion exercises. One study [36] compared MET plus exercise with a sham treatment of passive upper and lower limb stretching plus the same exercise program as the intervention group.

Two studies reported the specific muscles treated including the scalenes, sternocleidomastoid, upper trapezius and pectoralis major muscles [36, 37]. The other study [35] only described the movement that was resisted. The number of treatments varied, one study administered only one treatment, [35] another study included three sessions (one per day for three consecutive days) [37] and the other study performed 24 treatments (two per week for 12 weeks) [36].

**Fig. 2:**
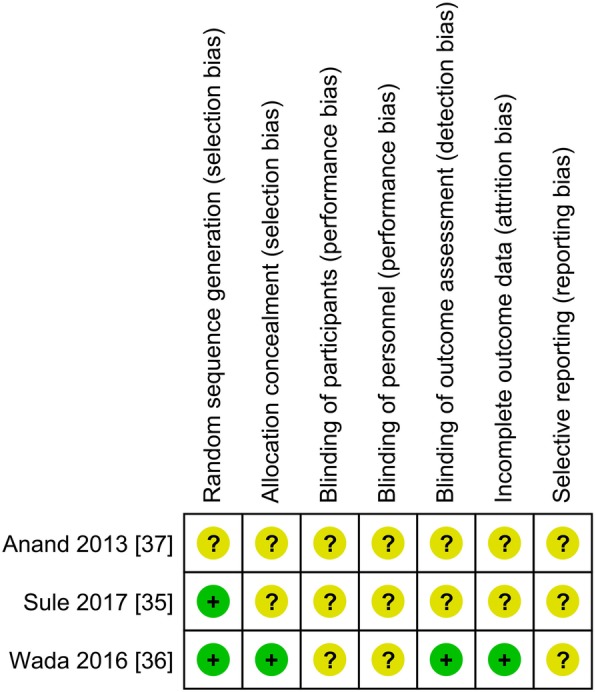
Risk of bias summary

The included studies were at risk of bias and had limitations in reporting quality. Two studies reported adequate random sequence generation [35, 36]. One study used an appropriate method of allocation concealment, [36] and the other studies were judged at unclear risk of bias because they omitted details relating to allocation concealment [35, 37] (Fig. 2). In two studies, blinding of participants and personnel was not described despite the use of a sham control. As blinding was not described and it was unclear whether a lack of blinding would influence outcomes, the study was judged as unclear risk for blinding of participants. Only one study specified that assessors of outcome measures were blinded. [36] None of the studies had published protocols, therefore, the selective outcome reporting domain was judged at unclear risk.

Two studies assessed FEV_1_, [35, 36] and one reported data suitable for analysis [35]. The study did not specify whether the FEV_1_ was presented as litres or percentage predicted, however, based on the data it was considered most likely to be percentage predicted. Results showed that MET plus CPT was not superior to sham plus CPT at the end of treatment (MD 4.87, 95% CI − 0.79 to 10.53).

Two studies assessed exercise capacity using the 6MWT [36, 37]. Individual study results showed that MET plus CPT was superior to CPT alone (153.47 m, 95% CI 110.48 to 196.46) [37]. In the other study, MET plus exercise therapy administered twice weekly for 12 weeks improved walking distance compared to sham-MET plus exercise therapy (34 m, 95% CI 21.07 to 46.93) [36].

Two studies reported perceived dyspnoea using the Borg and modified Borg scales [36, 37]. Although these results could not be pooled for meta-analysis, both showed reduced dyspnoea after MET. When MET plus CPT was compared with CPT alone, the Borg score was 1.46 points lower in the intervention group (95% CI − 1.91 to − 1.01) [37]. When MET plus exercise was compared with sham-MET plus exercise the modified Borg score was 1.25 points lower in the intervention group (95% CI − 1.48 to-1.02) [36].

One study reported health status using the Clinical COPD Questionnaire (CCQ) [37]. In people who received MET plus CPT the CCQ score at end of treatment was 0.93 points lower than people who received CPT alone (95% CI − 1.41 to − 0.45). Other outcomes such as inspiratory capacity and forced vital capacity were not assessed in the included studies.

One study reported adverse events, including one case of renal calculus exacerbation in the intervention group and one case of exacerbation of Crohn’s disease in the control group [36]. The study did not specify the severity of these events, though as they are both systemic in nature it is unlikely they were related to the intervention. Both participants dropped out of the study. The other two studies did not report adverse events [35, 37].

## Discussion

There are few studies that have evaluated this intervention. Diversity in treatment protocols of included studies meant that meta-analyses could not be performed. However, preliminary results from single studies showed favourable effects of MET compared to CPT and sham-MET in improving exercise capacity, dyspnoea ratings and health status. Due to the small number of studies and participants, differences in methodology, MET treatments, outcome measures, and unclear risk of bias, the potential benefits should be considered with caution. Only one study reported adverse events and the full extent of MET safety is unclear.

All included studies were published between 2013 and 2017, suggesting that the use of MET for COPD is a relatively new area of research. The studies were homogeneous in terms of participant population, including those with a diagnosis of moderate to severe COPD aged over 40 years. Conversely, there was heterogeneity in the application of the intervention and outcomes assessed.

Walking distance increased after MET treatments in two studies [36, 37]. Both studies reported differences in exercise capacity that were clinically important, with distances similar to- or exceeding- the minimum clinically important difference of 25–35 m [38]. This result was consistent with other studies of manual therapies for COPD that showed improvements in exercise capacity [16, 18, 39, 40].

Interestingly, improvement in the perceived dyspnoea ratings were shown in the same studies that showed improvement in exercise capacity [36, 37]. In the study by Wada et al. [36] a mean difference of 1.25 points between groups was reported*.* This exceeds the minimum clinically important difference for the modified Borg of one point [41]. This is consistent with other manual therapy studies which showed improvements in dyspnoea ratings after treatment [19, 39]. Clinically this may be relevant as dyspnoea is a known limiting factor to exercise capacity in this population [8, 9].

In terms of lung function, results from this systematic review are consistent with findings from a recent review of manipulative therapies for COPD that concluded the addition of manual therapy does not have any effect on lung function [42]. However, there are some preliminary findings in smaller studies not included in this review that show potential improvements in vital capacity [31] and inspiratory capacity [16, 17].

The minimum clinically important difference for the CCQ is 0.4 points [43]. The difference seen between groups in the CCQ score exceeded this. Large variation in the confidence intervals was found for this outcome and the 6MWD. When considered in relation to the small number of MET treatments applied, the results should be interpreted with caution.

Adverse events were reported in only one of the three studies. Wada et al. [36] reported one adverse event in both the control and intervention group which caused the withdrawal of two participants. The events occurred in the renal and gastrointestinal system and are unlikely to be related to the intervention. The lack of adverse event reporting in these studies may be partially attributed to the general confusion in the literature surrounding what constitutes an adverse event in manual therapy. The definition of adverse events in manual therapy can have a wide scope, depending on the context when it occurred. Factors include whether it is seen to be related to the intervention or whether it was an isolated incident in activities of daily living; perceptions of the patient and practitioner regarding the severity and impact of the incident [44]. It is generally accepted that minor adverse events may occur more commonly with manual therapy interventions, and as such are expected to be transient, short term incidents that do not require further treatment. [45] Examples of such incidents include post-treatment muscular soreness, headaches, and light headedness.

Several limitations of this review are acknowledged. Variance in treatment protocol between studies is likely to have influenced results. The number of treatments varied considerably between studies, though no clinical guidelines exist to guide treatment frequency or duration. Another differentiating factor may be that the application of MET in included RCTs was not typical of how it is used in clinical practice by manual therapists. It is also acknowledged that MET used as a component of an overall treatment plan, in combination with other techniques, may produce different results. Although MET used in isolation does not necessarily reflect clinical practice, it is important to ascertain the safety profile of MET treatment when used as an adjunct to standard care in individuals with COPD.

The sample size in all studies was small. One study did not report the number of people in each group. The authors of this study were contacted multiple times, with no response received. The assumption of equal numbers per group may have resulted in over or under estimation of the treatment effect. The narrow scope of this review, focussing on MET alone or as an adjunct to COPD treatments, identified few studies. Only one of the studies reported adverse events and there is insufficient evidence on the safety of MET for people with COPD.

Further rigorous research is required to ascertain the clinical efficacy and safety of MET for people that have COPD. Studies should be clear in the reporting of methodology, pre and post intervention outcome measures and demonstrate transparency in reporting of statistical analyses. Studies should also be clear in the reporting of treatment protocols to allow for study replication that can be translated into clinical practice. There is potential for mechanistic studies to be completed for further elucidation of manual intervention in any population.

## Conclusion

From this review, there is insufficient evidence to inform or support the use of MET for COPD. Included studies are small and at an unclear risk of bias. Results from three single studies showed potential benefits for improvements in outcomes such as exercise capacity and dyspnoea. However, rigorously designed research is needed to further examine the potential role of MET used in the management of COPD. Practitioners should use their clinical judgement about the suitability of this intervention based on individual patient presentation.

## Additional file

Additional file 1: Muscle Energy Technique for COPD – PRISMA 2009 checklist, Word document. Describes location of PRISMA reporting items in manuscript text. (DOC 63 kb)

## Abbreviations

6MWT: Six-minute walk test
CCQ: Clinical COPD Questionnaire
COPD: Chronic Obstructive Pulmonary Disease
CPT: Conventional Chest Physiotherapy
FEV: Forced expiratory volume
GOLD: Global Initiative for Chronic Obstructive Lung Disease
MET: Muscle Energy Technique
RCT: Randomised controlled trial
